# Simplifying Iliotibial Band Lateral Extra‐articular Tenodesis Fixation During Anterior Cruciate Ligament Reconstruction: A Hardware‐Sparing Approach

**DOI:** 10.1002/atn2.70138

**Published:** 2026-06-08

**Authors:** Emmanouil Papakostas, Enejd Veizi, Ashraf Hantouly, Aizel Sherief Palasseril, Merve Bozer Kavukcu, Alan Getgood

**Affiliations:** ^1^ Surgery Department Aspetar Orthopaedic and Sports Medicine Hospital Doha Qatar; ^2^ Department of Orthopedic Surgery McMaster University Ontario Canada; ^3^ Department of Orthopedics and Traumatology Kaman State Hospital Kirsehir Turkey

## Abstract

The addition of a lateral extra‐articular tenodesis to anterior cruciate ligament reconstruction has been shown to reduce graft failure and improve rotational stability, but current fixation methods often require additional tunnels or hardware, with risks of tunnel convergence, irritation, and hardware removal. This technical paper describes a modified Lemaire lateral extra‐articular tenodesis technique in which the iliotibial band flap is secured directly to the femoral suture button of the anterior cruciate ligament graft, thereby avoiding additional fixation devices. This technique minimizes the risk of tunnel convergence, leaves a smoother lateral condylar surface, and reduces postoperative irritation associated with metallic fixation, while providing strong fixation in the isometric zone.

## Graphical abstract

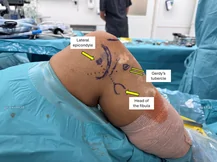

**VIDEO 1:** A small lateral incision is made to harvest an 8‐10 mm wide, 8‐10 cm long iliotibial band flap that remains distally attached at the Gerdy's tubercle. Arthroscopic anterior cruciate ligament reconstruction is then performed in standard fashion. After tibial and femoral fixation of the anterior cruciate ligament graft, attention shifts laterally, where the button's running and adjusting sutures are retrieved and used to tension and secure the iliotibial band flap, eliminating the need for additional lateral extra‐articular tenodesis‐specific implants. One running suture is passed in a locking fashion through the proximal portion of the flap, while the second acts as a shuttle to draw the flap directly over the button. The sutures are tied at 60° of flexion to avoid over‐constraint, and the adjusting sutures are subsequently tied over the flap to reinforce the construct. The knee is cycled through its full range of motion to ensure smooth tracking before final closure. Video content can be viewed at https://doi.org/10.1002/atn2.70138.

The addition of lateral extra‐articular procedures after a primary or revision anterior cruciate ligament reconstruction surgery (ACLR) is an ever‐increasing phenomenon in the field of sports surgery.^1^, ^2^, ^3^, ^4^ The goal is to manage high‐grade anterolateral rotational instability after an ACLR.^5^, ^6^ The rationale for such a concomitant procedure has been proven by several publications and is known to decrease overall revision rates as well as providing additional stability after ligament reconstruction with augmented rotational stability, especially when using hamstring autografts, in risk population and activities.^2^, ^5^, ^7^, ^8^, ^9^, ^10^

The modified Lemaire lateral extra‐articular tenodesis (LET), one of the most frequently preferred procedures, consists in the fixation of a section of the iliotibial band (ITB) to the cortex of the lateral condyle, thus mimicking the role of an injured anterolateral complex.^7^, ^11^, ^12^, ^13^ The procedure has been shown to be clinically well tolerated with reduced revision rates and lower rates of osteoarthritis in the long term.^14^, ^15^ Several fixation methods for LET have been described in the literature with different biomechanical properties.^16^, ^17^ Blind tunnels with inlay biodegradable screw fixation, although biomechanically robust, are challenging in the setting of an already violated lateral condyle. LET tunnel convergence with the ACL graft is a fairly common complication.^11^ An alternative fixation modality are suture anchors and onlay staples.^1^, ^5^, ^16^ However, irritation on the surgical site due to hardware protrusion and reoperation for hardware removal is not an uncommon occurrence,^18^, ^19^ having also the risk of compromising femoral ACL tunnel.

The aim of this article is to describe an alternative fixation modality for securing the LET at the cortex of the lateral condyle.

## SURGICAL TECHNIQUE

**FIGURE 1:**
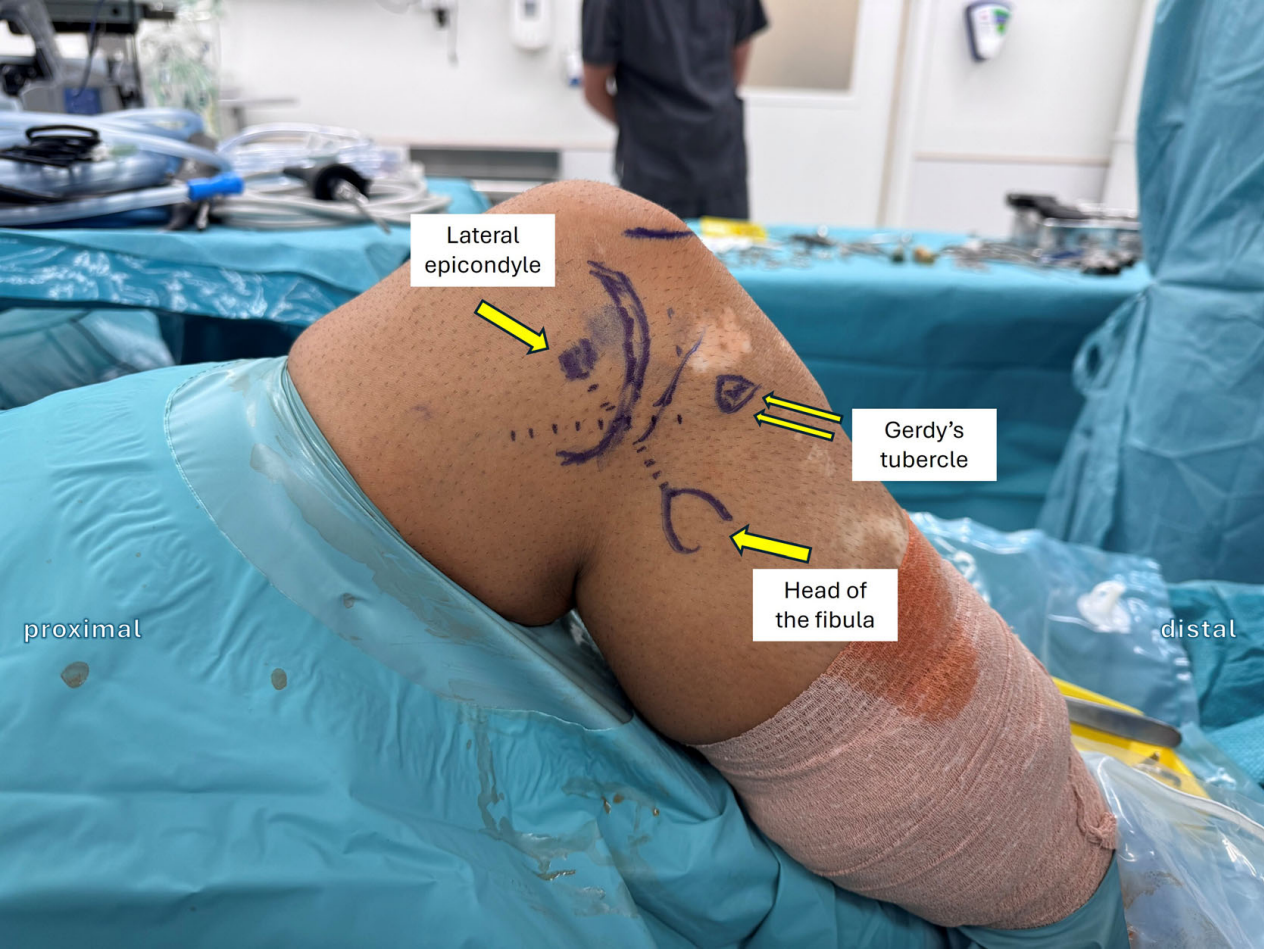
The lateral condyle (patient supine, right knee), the fibular head and the Gerdy's tubercle are marked and an incision of approximately 5‐8 mm is performed between the upper 2/3 and the lower 1/3 border of the lateral condyle, distally aimed towards the Gerdy's tubercle.

**FIGURE 2:**
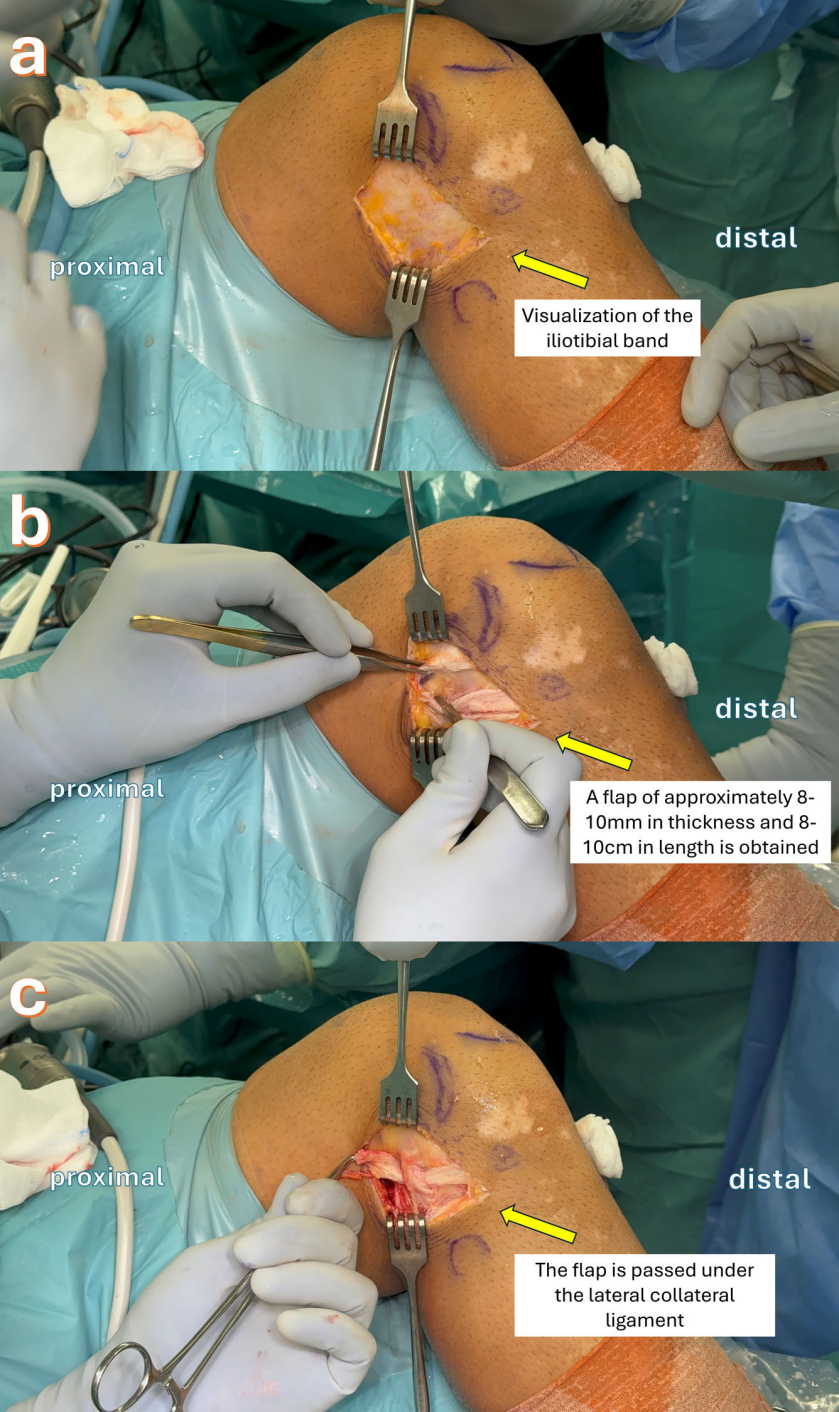
(a) Skin flaps are elevated and the iliotibial band is visualized (patient supine, right knee). (b) An iliotibial band flap of approximately 8‐10 mm in thickness and 8‐10 cm in length is created for the lateral extra‐articular tenodesis procedure, and the flap is released proximally but is kept intact distally at the Gerdy's tubercle. (c) The created flap is then buried into the tissues, and the attention is shifted to the arthroscopic procedure.

The patient is positioned supine on the surgical table, and a tourniquet is placed on the proximal thigh of the indexed lower extremity and inflated (Video 1). After skin preparation, the lateral condyle is marked and an incision of approximately 5‐8 mm is performed between the upper 2/3 and the lower 1/3 border of the lateral condyle, distally aimed towards the Gerdy's tubercle (Figure 1). Skin flaps are elevated and the ITB is visualized. Its lower border is identified, and a longitudinal parallel incision is performed approximately 10 mm above it. This is followed by a second parallel incision from the first one, creating an ITB flap of approximately 8‐10 mm in thickness and 8‐10 cm in length. The created flap is released proximally but is kept intact distally at the Gerdy's tubercle. The created flap is then buried into the tissues, and the attention is shifted to the arthroscopic procedure (Figure 2).

The knee joint is accessed using standard anteromedial and anterolateral arthroscopic portals. After a thorough diagnostic arthroscopy, the rupture of the ACL is confirmed, and the remnant is debrided. The autograft is then harvested and prepared. The graft's length and diameter are subsequently measured. All types of autografts are eligible for this technique, as long as an adjustable button is used for the femoral fixation. While the graft is being prepared for fixation, the femoral tunnel entry point is marked and then drilled at the site of the anteromedial bundles of the native ACL. Both antegrade and retrograde drilling can be used for femoral socket creation, with the appropriate diameter. The exit point of the tunnel is aimed to be posterior and proximal to the lateral epicondyle and is checked through palpation or direct vision from the already open lateral incision. In case the exit point is different from the original plan during antegrade pin placement, knee flexion can be increased/decreased so that the angle of the guiding pin can be adjusted intra‐articularly, until the optimal position is achieved. The tunnel is then drilled, and a passing suture is advanced.

The tibial tunnel is then prepared in standard fashion, with an intra‐articular aim on the native fibers of the ACL remnant, 3‐5 mm medial to the anterior horn of the lateral meniscus. After adequate drilling, retrograde or antegrade according to the preferred technique and/or graft, the graft is then advanced in both tunnels and fixed with an adjustable loop system over a button (depending on the surgeon's preference in our institution, Smith & Nephew, ConMed, Arthrex or Biotek) for femur and the surgeon's preferred method in tibia (adjustable button, interference screw with or without secondary fixation, etc.).

**FIGURE 3:**
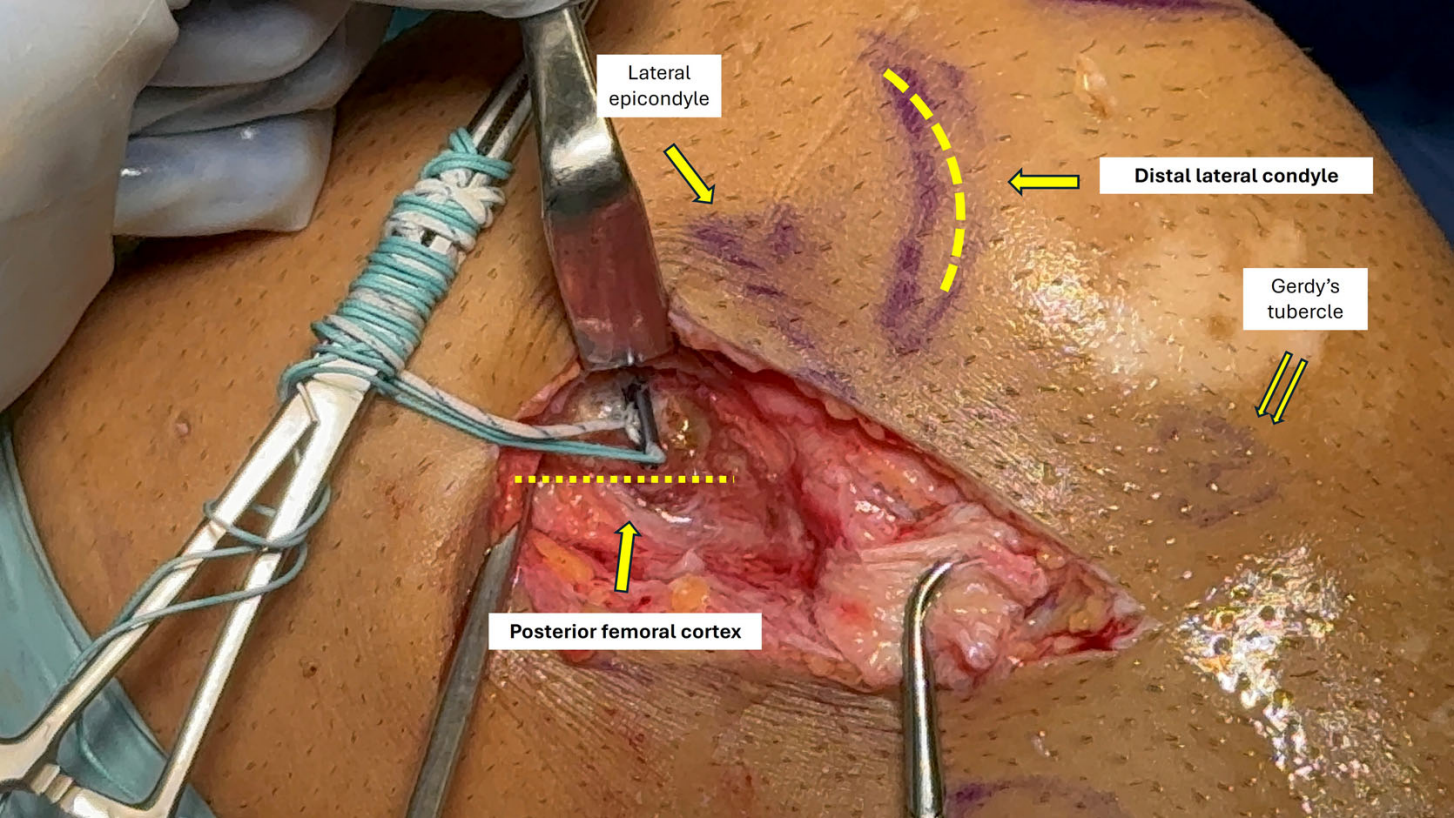
The exit point of the anterior cruciate ligament's femoral tunnel and the adjustable suture button's location is then found, and the anatomical landmarks are again identified to confirm optimal position (patient supine, right knee). The adjusting sutures as well as running sutures of the flipping mechanism are retrieved from the open incision.

**FIGURE 4:**
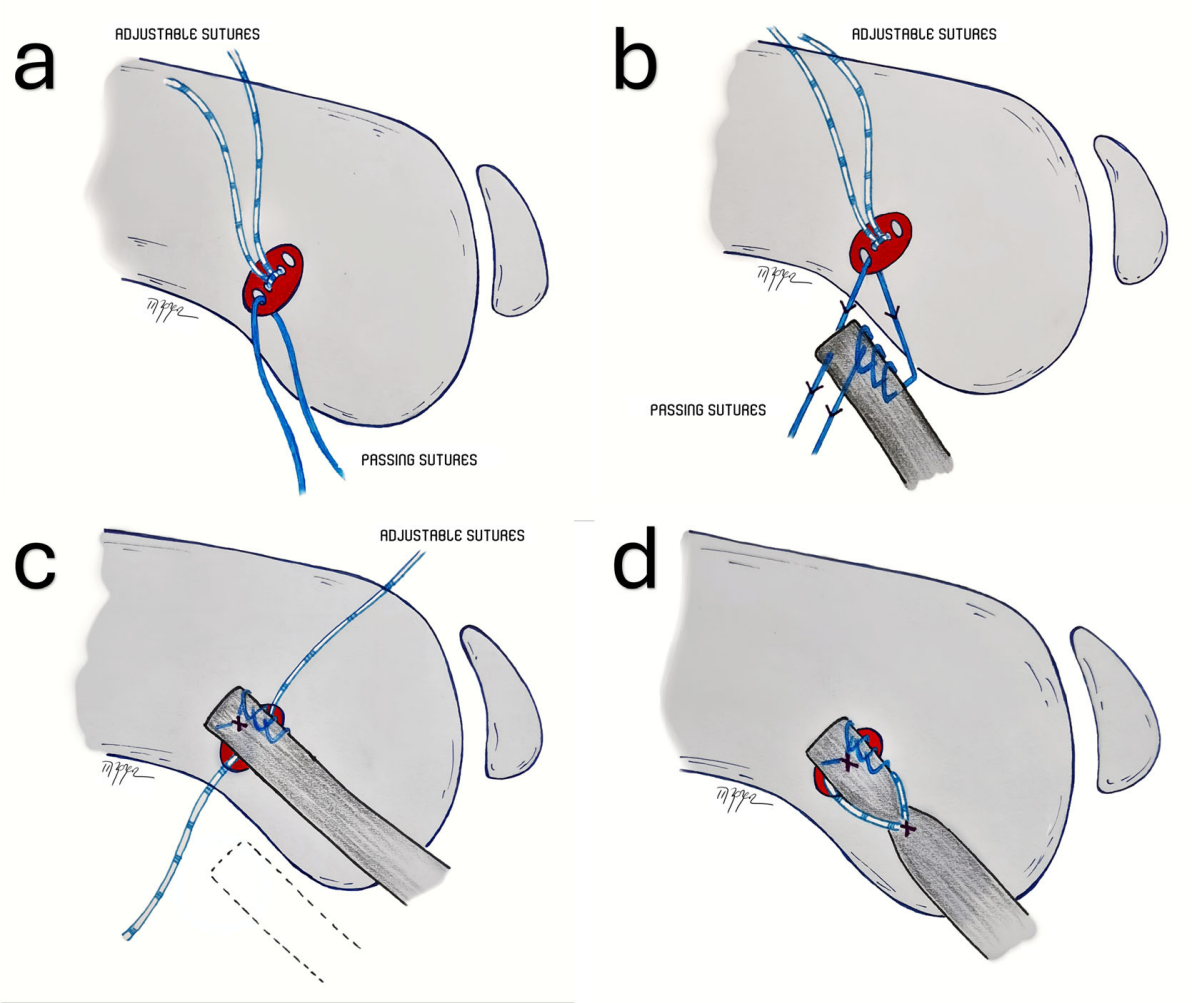
(a) One of the running sutures is passed through the marked proximal part of the iliotibial band flap with a free needle and in a locking fashion. (b) The other running suture is also passed through the flap only once, thus creating a shuttling mechanism which will pull the iliotibial band flap directly over the suture button. (c) The adjusting sutures are then, (d) in turn, knotted over the newly fixed iliotibial band flap, always distal to the first knot, providing additional, secondary fixation.

**FIGURE 5:**
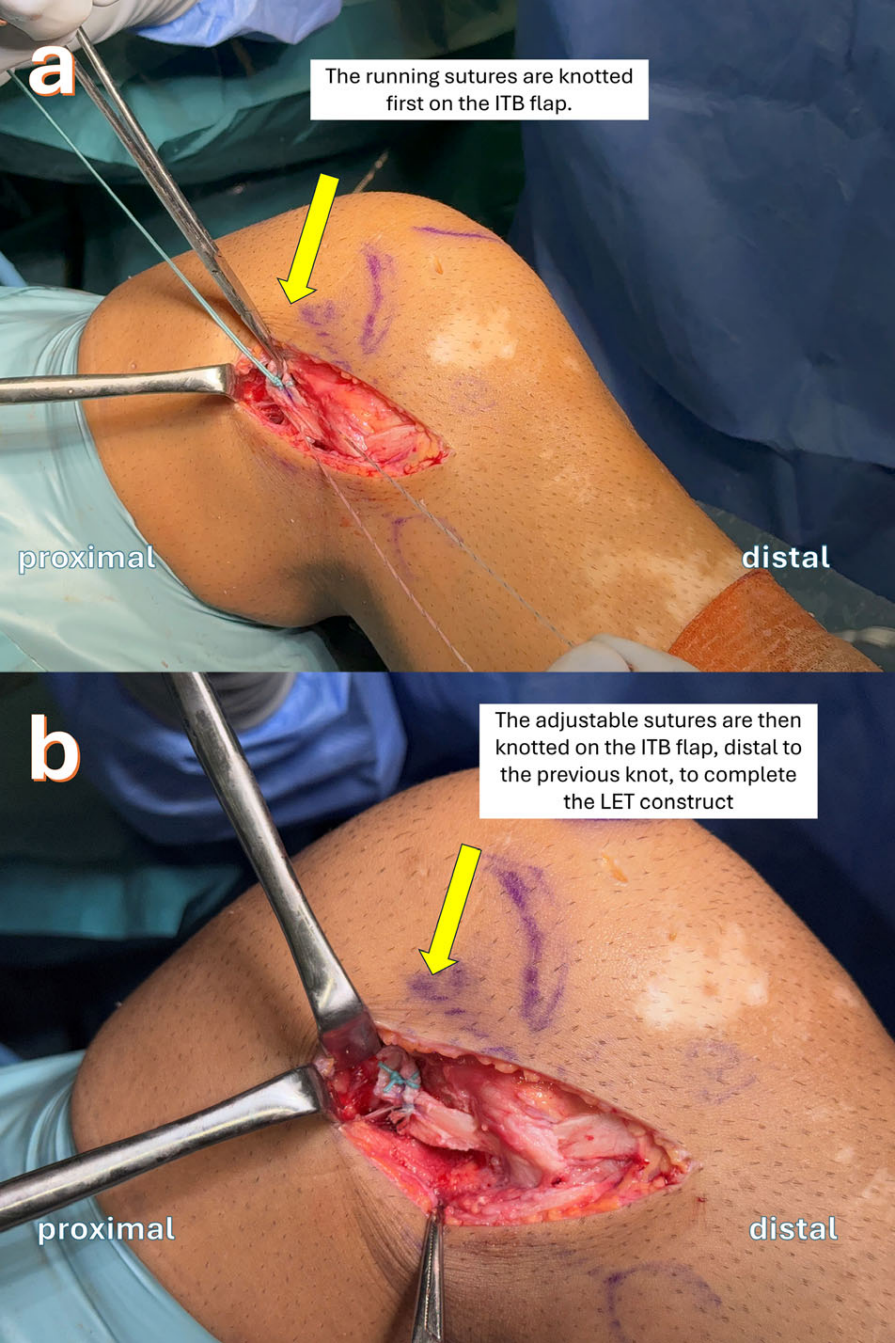
(a) The running sutures are knotted over the button at 60° of knee flexion and neutral tibia rotation, followed by the adjusting sutures, slightly distal (patient supine, right knee). (b) The final construct is clinically efficient, well tolerated by the patients and with less irritation on the fixation site. (ITB, iliotibial band; LET, lateral extra‐articular tenodesis.)

Attention is then drawn to the lateral extra‐articular procedure. The exit point of the ACL's femoral tunnel and the adjustable suture button's location is then found, and its adjusting sutures as well as running sutures of the flipping mechanism are retrieved from the open incision (Figure 3). A starting point for the suturing is decided and marked with the knee in extension to avoid over‐tensioning in full range of motion. One of the running sutures is then passed through the marked proximal part of the ITB flap with a free needle and in a locking fashion. The other running suture is also passed through the flap only once, thus creating a shuttling mechanism which will pull the ITB flap directly over the suture button (Figure 4). The running sutures are knotted over the button at 60° of knee flexion and neutral tibia rotation. The adjusting sutures are then, in turn, knotted over the newly fixed ITB flap, always distal to the first knot, providing additional, secondary fixation (Figure 5). The knee is then run through the whole range of motion. The ITB is then approximated, and the remaining skin flaps are closed in standard fashion.

In the absence of meniscal lesions, postoperative rehabilitation the following day consists of assisted weight bearing as tolerated, range of motion, and closed chain exercises.

## DISCUSSION

The addition of a LET after a primary ACL is an increasingly popular trend. In their original study, Getgood et al.^5^ showed that the addition of a LET to an ACLR with hamstring autografts led to lower revision rates and less overall clinical failures. More recent literature has built upon these findings, showing that lower graft failure rates, reduced anteroposterior laxity, and diminished high‐grade pivot shift were additional advantages to the addition of LET offered after an ACLR.^1^, ^3^, ^8^, ^14^, ^18^ The discussion is still open, on whether this leads to a quicker return to sport or to higher patient‐reported outcome measures.^2^, ^15^

A lack of consensus exists among the orthopaedic community with regard to the method of fixation for the LET component and indeed on the best point of fixation and the best degree of flexion.^3^, ^11^ Studies have reported results with fixation achieved though suture anchors, onlay staples, and screws,^16^, ^19^ and although most of these fixation methods have been shown to be well tolerated, there are still complications. The main concern is tunnel convergence with the ACL femoral tunnel, which is especially close to the attaching point of the Lemaire technique.^11^ This convergence rarely seems to happen mid‐tunnel as it has been shown to occur at the exit point on the lateral condyle, thus posing a risk for fixation failure and need for early revision. The technique mentioned in this study aims to avoid the occurrence of convergence altogether, leading to a similarly good fixation combined with a smother overall construct (Tables 1 and 2).

**TABLE 1:** Pearls and Pitfalls for the Described Technique

**Pearls**
• Approaching first the lateral extra‐articular tenodesis is of a great advantage since it allows to control the exit point of the ACL as well as helps avoid swelling of the tissues through extravasation. • If a conventional, arthroscopy‐first approach is to be followed, make sure, through skin palpation, the ACL's femoral tunnel exit point is between the lateral epicondyle and the posterior margin of the lateral femoral condyle. This can be fine‐tuned by changing the angle of knee flexion during guiding pin placement, to achieve an optimal exit point. • If needed, an radiograph could be contemplated to confirm the tunnel exits proximally and posterior to the lateral epicondyle, close to the isometric area. • After dissection of the surrounding soft tissues, the metal button might loosen on the femoral condyle. Make sure the adjustable loop is well tightened again, before pulling the ITB flap towards it. A strong fixation of both the graft and the LET procedure should be obtained at the end of this technique.
**Pitfalls**
• Although the isometric area is quite wide, failure to exit posterior and proximal to the lateral condyle will lead to an anisometric lateral extra‐articular tenodesis. In this case, the fixation of the ITB flap could be performed through an additional anchor. • Avoid cutting the adjustable loop of the metal button holding the ACL's graft while cutting the final sutures.

ACL, anterior cruciate ligament; ITB, iliotibial band; LET, lateral extra‐articular tenodesis.

**TABLE 2:** Advantages and Limitations of the Technique Described

**Advantages**
• The procedure does not require an additional tunnel for the fixation of the ITB as a lateral extra‐articular procedure. • The fixation is strong, and the LET is placed in the isometric area. • A smoother area is left on the lateral condyle, thus avoiding irritation on palpation and requiring no hardware removal.
**Limitations**
• A misplaced femoral tunnel will lead to an inappropriate fixation site for the LET. • The knot used for the fixation of the ITB flap, if overdone, could lead to irritation on the tenodesis site.

ITB, iliotibial band; LET, lateral extra‐articular tenodesis

Onlay metallic staples are an additional method of fixation for the LET.^17^ They offer the advantage of avoiding tunnel convergence during rigid time‐zero fixation.^16^ They can also be used in pediatric patients, without fear of physeal injury. Despite these features, irritation on the fixation site is a well‐known complication of metallic staples often requiring a reoperation for hardware removal.^19^ The present technique, although it has not been investigated in a biomechanical setting, offers the possibility of a smoother surface area on the fixation site while still securing the proximal end of the ITB to the lateral femoral condyle and creating a “closed loop of fixation” as in the setting of an over‐the‐top technique, where the forces and implants holding the ACL graft also hold the LET construct. Similar to other inlay fixation methods, such as metal or suture anchors, the ITB is secured with reinforced sutures in an area roughly between the Lemaire and MacIntosh points. Moreover, this technique is fairly reproducible and compatible with all kinds of autografts and allografts. The fixation at the suture button's site is suitable for anterograde, retrograde femoral tunnel drilling, and for all kinds of ACLR techniques.

This alternative technique can be limited by a couple of factors. A misplaced femoral tunnel could lead to an inappropriate fixation site for the LET. We suggest that the surgeon always double check the intra‐articular as well as the exiting point of the femoral tunnel before deciding to proceed with drilling, as this would limit the ability of the LET to provide extra rotatory stability. Additionally, the knot used for the fixation of the ITB flap, if overdone, could lead to irritation on the tenodesis site. Despite these potential limitations, the technique does not require an additional tunnel or hardware for the fixation of ITB as a lateral extra‐articular procedure.

In conclusion, the technique described offers an alternative way of augmenting the ACLR with a lateral extra‐articular procedure that is clinically efficient, well tolerated by the patients and with less irritation on the fixation site. Clinical outcomes derived from larger cohorts involving patients treated with this technique are imperative to make further conclusions.

## DISCLOSURES

The authors (E.P., A.G.) declare the following financial interests/personal relationships which may be considered as potential competing interests: E.P. reports a relationship with Smith & Nephew, Arthrex (biologics), ConMED, and Biotek that include: consulting fees, support for attending meetings and/or travel; reports a relationship with Geistlich and Anika that include: payment or honoraria for lectures, presentations, speakers bureaus, manuscript writing or educational events; reports a relationship with Askel Healthcare that includes: participation on a Data Safety Monitoring Board or Advisory Board; reports a relationship with ICRS (Secretary General—Member EB) that includes: leadership or fiduciary role in other board, society, committee or advocacy group, paid or unpaid. A.G. receives the research support from NIH and CIHR; reports a relationship with Smith & Nephew that includes: royalties or licenses, consulting fees, payment or honoraria for lectures, presentations, speakers bureaus, manuscript writing or educational events, payment for expert testimony, support for attending meetings and/or travel, patents planned, issued or pending; reports a relationship with Precision OSK, Kyniska Robotics, Spring Loaded Technology that include: other financial or non‐financial interests. The other authors (E.V., A.H., A.S.P. M.B.K.) declare that they have no known competing financial interests or personal relationships that could have appeared to influence the work reported in this paper.
